# Investigating the psychometric properties of PaRCADS—Parenting to Reduce Child Anxiety and Depression Scale in a Norwegian sample

**DOI:** 10.1002/mpr.2017

**Published:** 2024-03-09

**Authors:** Kristin Ytreland, Jo Magne Ingul, Stian Lydersen, Marie Bee Hui Yap, Wan Hua Sim, Anne Mari Sund, Elisabeth Valmyr Bania

**Affiliations:** ^1^ Regional Centre for Child and Youth ‐ Mental Health and Child Welfare (RKBU), Central Norway Faculty of Medicine and Health Science Department of Mental Health Norwegian University of Science and Technology (NTNU) Trondheim Norway; ^2^ School of Psychological Sciences Turner Institute for Brain and Mental Health Monash University Melbourne Victoria Australia; ^3^ Melbourne School of Population and Global Health University of Melbourne Melbourne Victoria Australia

**Keywords:** anxiety, depression, PaRCADS, parenting, psychometrics

## Abstract

**Objectives:**

Parents play a pivotal role in child development and several parental factors have been identified as risk or protective factors for childhood anxiety and depression. To assess and target these parental factors in interventions, there is a need for a comprehensive, easy‐to‐use instrument.

**Method:**

This study aimed to investigate the psychometric properties of an adapted version of the Parenting to Reduce Child Anxiety and Depression Scale, PaRCADS(N) in a Norwegian community sample (*N* = 163) of parents of children aged 8–12 years.

**Results:**

Our findings indicate that PaRCADS(N) has acceptable psychometric properties. These results are comparable to those of the original study of the PaRCADS in Australia.

**Conclusion:**

Based on these results, we recommend that PaRCADS(N) can be utilized by health care workers as a tool for assessment and identification of parental practices related to child anxiety and/or depression to target relevant risk and protective factors in treatment and prevention.

## 1 INTRODUCTION

Anxiety and depression are relatively common in children and adolescents. A meta‐analysis of prevalence studies found that the global prevalence rate for anxiety in children and adolescents between 4 and 18 years old was 6.5% and for depression 2.6%. In a recent Norwegian, community‐based longitudinal study, Steinsbekk et al. (2022) and Morken et al. (2021) found that the prevalence of anxiety disorders in children ranged from 0.7% at age 4 to 5.3% at age 14 with a substantial increase noted in children between the ages of 8 and 10. At the age of 14 nearly 10% of all child participants had experienced anxiety disorder (Steinsbekk et al., 2022). In the same study population, the prevalence of major depressive disorder ranged from 0.1% at age 4 to 2.7% at age 14, with a substantial increase after the age of 12 (Morken et al., 2021).

Child anxious and depressive problems may pose a risk for later disorders, and lead to impairments in several aspects of life, such as school functioning, social relationships, life satisfaction, and work life, such as late entry into the workforce, and poorer work performance (Balazs et al., 2013; Clayborne et al., 2019; Swan & Kendall, 2016). Thus, left untreated, these problems can prove costly for the individual, their family, and society; through healthcare costs, lack of income, and productivity loss (Trautmann et al., 2016).

Parents play a pivotal role in child development and wellbeing, and robust evidence have highlighted various parental factors associated with the development and maintenance of child anxiety and depression, as risk or protective factors (Pinquart, 2017; Yap & Jorm, 2015). Some factors are difficult to modify, such as socioeconomic status or poverty, or an inherited genetic predisposition for mental disorders (Marees et al., 2021). However, some risk‐factors associated with child and adolescent anxious and depressive problems, such as inter‐parental conflict, controlling parental behavior or over involvement (Bayer et al., 2019; Siqueland et al., 1996; Westrupp et al., 2018) are behavioral and more malleable. Thus, targeting these parental factors, while amplifying protective factors, such as a positive parent‐child relationship (Wu & Lee, 2020), will likely enhance the efficacy of prevention or treatment interventions for childhood anxiety and depression. To successfully target these parental factors, the first step is to identify areas in which the parent has potential for improvement.

A simple and cost‐effective method could be a validated and evidence‐based instrument for mapping the parental factors in question. Such an instrument may improve assessment before an intervention as well as aid planning and monitoring of the parent's development during and after the intervention.

The Parenting to Reduce Child Anxiety and Depression Scale (PaRCADS) (Sim et al., 2019) is a parent self‐report instrument developed in Australia to assess various domains of parenting associated with childhood anxiety and depression. As a criterion‐referenced measure, it was designed to measure parents' concordance with parenting guidelines for the prevention of childhood depression and anxiety (Parenting Strategies Program, 2014). These guidelines were developed based on a systematic review of literature on important parental risk and protective factors for childhood anxiety, depression and internalizing problems (Yap & Jorm, 2015) and a Delphi study of international expert consensus (Yap et al., 2015). PaRCADS consists of 79 items, across 10 domains: *Relationship with your child, Involvement in your child's life, Child's relationship with others, Rules and consequences, Health habits, Home environment, Managing emotion, Setting goals and dealing with problems, Dealing with negative emotions, and Getting help when needed*. Results are originally scored as 0 = non‐concordant or 1 = concordant with the Parenting Strategies Program's (2014) parenting guidelines. Higher scores indicate more positive parental practices.

In one Australian community‐based study (Sim et al., 2019) PaRCADS showed acceptable psychometric properties and construct validity as indicated by adequate correlations with similar well‐established measures: the Parent report of the Children's Report of Parent Behavior Inventory (P‐CRPBI) Acceptance subscale (Schaefer, 1965) (*r* = 0.54) and the Family Assessment Device (FAD) General Functioning subscale (Epstein et al., 1983) (*r* = −0.52). The International Test Commission (2017) published guidelines for translation and adaptation of tests, such as psychometric instruments. These guidelines highlight the importance of careful adaptation to the new language and culture while remaining true to the original. Following these guidelines, PaRCADS was translated and adapted to Norwegian culture, and named PaRCADS(N). Hence, it is important to establish PaRCADS(N)'s psychometric properties to ensure that this new version measure the same constructs as originally intended.

In this study, we tested whether PaRCADS(N) is suitable as a norm‐referenced scale, rather than a criterion‐referenced scale. This allows for larger variance and ability to monitor even smaller changes over time, for instance, before, during, and after an intervention.

### 1.1 Aims and hypotheses

The aim of this study was to investigate the psychometric properties of PaRCADS(N) in a community sample of Norwegian parents, by establishing its reliability, validity, and factor structure. To assess whether PaRCADS(N) is similar to the original PaRCADS, we compared our results to those of the original PaRCADS study, the methods are described in Sim et al. (2019).

We hypothesized that PaRCADS(N) demonstrates 1. Good internal consistency and test–retest reliability. 2. Adequate construct validity, as the compared instruments measure similar, but not identical constructs. Specifically, we predicted that the Parental Bonding Instrument (PBI), *Warmth* dimension would have higher correlations with the PaRCADS total score and domains pertaining to parent‐child relationship and parental warmth; *Relationship with your child, Involvement in your child's life, Managing emotions, Setting goals and dealing with problem*s, *Dealing with negative emotions*, and less with other domains which measure other parental constructs, not related to parent‐child relationship or parental warmth such as *Health habits*. We further predicted a moderate to strong negative correlation between PaRCADS(N) total score and general family functioning (as measured by the FAD, General Functioning subscale; FAD‐GF) as higher scores in FAD‐GF indicates poor family functioning. 3. A small negative correlation with parent‐reports of child anxiety and depression symptoms as PaRCADS was designed to map parental practices related to the development and maintenance of childhood anxiety and depression 4. Adequate to good model fit. A confirmatory analysis would not show a perfect fit of the model as the criterion referenced PaRCADS was not designed with this in mind. However, we predicted an adequate to good model fit, as PaRCADS domains are based on previous research on parental factors associated with childhood anxious and depressive problems.

An exploratory analysis was undertaken to explore differences in PaRCADS results between this sample and the original PaRCADS‐study in Australia (Sim et al., 2019), there was no sufficient evidence to make a directional hypothesis.

## 2 METHODS

### 2.1 Translation and adaptation

PaRCADS was translated and adapted to the Norwegian language and context, in line with the guidelines for translation and adaptation of tests (International Test Commission, 2017). Cultural adaptations such as phrasing were agreed upon with the original authors of the instrument (Sim et al., 2019), to ensure that the intended meaning was retained in PaRCADS(N). One example of such rephrasing was the term “misbehave”, which is not regularly used in daily speech in Norway. Thus, this was changed to “behaves badly”. Item 6.8 “I smack my child when I'm angry” was removed from the Norwegian version of the instrument as smacking your child is illegal in Norway and an affirmatory answer to this question would incriminate parents.

### 2.2 Procedure and participants

Parents were recruited to an online survey to evaluate PaRCADS(N), through schools' information channels such as electronic message systems and physical attendance at parent meetings. As an incentive to participate, 10 random participants (6%) received a gift card of 500 NOK (approximately 50 USD) after participation. Participants were 163 parents of 163 children aged 8–12 years. After participants provided informed consent in an online form, they were redirected to the survey. In addition to the PaRCADS(N), parents completed demographic questions for background information about themselves and their child, measures of childhood anxiety and depression symptoms, and established parent measures.

Participants were invited to complete a follow‐up survey to assess test–retest reliability. Response rate was 45% (*n* = 75). Parents completed the retest on average 87 days (SD = 35) after the initial survey. One parent was excluded from test–retest analysis as they answered the test–retest 238 days apart.

### 2.3 Measures

**The PaRCADS** (Sim et al., 2019), consists of 79 items across 10 domains. To assess its suitability as a norm‐referenced scale, we scored each PaRCADS item referring to parental behaviors on a 5‐point frequency scale, (0 = almost never to 4 = almost always), or a likelihood scale for hypothetical questions (0 = very unlikely to 4 = very likely). Higher scores reflect greater preventive parental practices. After we removed an item in the PaRCADS(N), the sum of possible scores in each domain ranged from 0 to 40 (dependent on number of items), the possible total scale score ranged from 0 to 312. Internal consistency reliability was generally adequate (McDonald's *ω* = 0.50–0.78), see Table 3.

**The Mood and Feelings Questionnaire—Short form (SMFQ)** (Angold et al., 1995), is a 13‐item parent‐report questionnaire designed to detect depressive symptoms in children aged 8–18 years. Responses are made on a 3‐point scale (0 = not true, 1 = sometimes true and 2 = true) (range 0–26). Mean SMFQ‐score in a Norwegian community sample of 10–19 year‐olds (*n* = 5804) was lower for boys (3.38, SD = 3.78) than girls (5.56, SD = 5.24) (Larsson et al., 2016). We therefore suggest that total scores of ≥7 (mean + one SD for boys) indicate elevated levels of depression symptoms. Good internal consistency has been found in both a Norwegian population study and a sample with elevated levels of anxious and depressive symptoms (Larsson et al., 2016; Martinsen et al., 2019). In this sample (*N* = 163) internal consistency reliability was good (*ω* = 0.86).

**The Screen for Child Anxiety Related Disorders (SCARED)** (Birmaher et al., 1997) is a 41‐item parent‐report questionnaire designed to detect symptoms of anxiety in children 8 years and older on five different subscales; *Panic/Somatic*, *Generalized*, *Separation*, *Social School avoidance* and a total score (range 0–82). Items are scored on a 3‐point scale (0 = not true or hardly ever true, 1 = somewhat true or sometimes true and 2 = Very true or often true). The original study (Birmaher et al., 1997) suggested threshold values for the instrument, with a total score of ≥25 indicates further investigation of an anxiety disorder. Ingul et al. (2012) reported good internal consistency of the scale, Cronbach's *α* = 0.62–0.88 for the subscales, and *α* = 0.94 for the total SCARED. In this sample (*N* = 163) internal consistency as calculated by McDonald's omega for the subscales (*ω* = 0.74 to *ω* = 0.89), and total scale (*ω* = 0.93) was comparable. We only used SCARED total score in this study.

**The PBI** (Parker et al., 1979), as revised and shortened by Kendler (1996) is a 16‐item parent‐report questionnaire designed to assess the parent‐child relationship on three dimensions; *Warmth* (range 7–28) which measures parenting characterized by positive emotions and communication, as well as two other dimensions not included in this study, *Protectiveness* and *Authoritarianism*. Items are scored on a 4‐point scale from 1 = fits very well to 4 = fits very poorly. We used the *Warmth* dimension, where a higher score on this subscale indicates more adaptive parenting, as a measure to examine the construct validity of PaRCADS. The psychometric properties of the Norwegian version of PBI *Warmth* dimension are adequate (*α* = 0.77) (Rimehaug et al., 2011). In this sample, internal consistency reliability of the warmth dimension was good (*ω* = 0.81).

**The General Family Functioning Subscale (FAD‐GF**) (Byles et al., 1988; Epstein et al., 1983) is a 12‐item parent‐report designed to assess the overall family functioning and is one of seven subscales in the FAD (Epstein et al., 1983). FAD‐GF measures families' overall healthy (6 items) and unhealthy functioning (6 items). Items are scored on a 4‐point scale from 1 = strongly agree to 4 = strongly disagree (range 12–48). A higher score indicates poorer family functioning, that is, poor emotional functioning and/or lack of practical organization between family members, and the original authors suggested a threshold of ≥24 (Epstein et al., 1983) to indicate poor family functioning. In this sample (*N* = 163) internal consistency reliability was good (*ω* = 0.87).

### 2.4 Statistical analysis

We used McDonald's omega as a measure of internal consistency of the scales used in this study. A more commonly used measure of internal consistency, Cronbach's alpha, assumes data to be normally distributed with equal variance and a good factor fit. McDonald's Omega is more robust if one or several of these criteria are not met, which is often the case in psychometric measures (McNeish, 2018). We considered internal consistency values of ≥0.7 as adequate, ≥0.8 = good, ≥0.9 = excellent (Evers et al., 2013). We examined reliability through test–retest and Pearson's correlation coefficient between participant subscales and total scores at two timepoints. For test–retest purposes, we considered values of *r* ≥ 0.6 as adequate, *r* ≥ 0.7 = good and *r* ≥ 0.8 = excellent (Evers et al., 2013).

Through PaRCADS(N)'s correlation with scores on instruments that measure similar or related constructs and have established psychometric properties, parent‐child relationship (PBI *Warmth*) and family functioning (FAD–GF) we assessed the construct validity of PaRCADS(N). We considered correlation coefficients ≥0.55 as adequate, ≥0.65 = good, and ≥0.75 = excellent (Evers et al., 2013). These thresholds should be interpreted with flexibility, as these three instruments measure similar, but not identical constructs.

In addition, we conducted a confirmatory factor analysis (CFA) to assess the hypothesized, predefined model; the relationship between indicators (observable variables), such as item responses, and identified factors (latent variables) such as parental factors underlying each domain of PARCADS (Brown, 2015). We included the 10 domains of PaRCADS as factors, and the 78 items as indicators, to investigate the theoretical factor structure of PaRCADS. The interpretation of CFA results are highly dependent on different cut‐off values of several fit indices. These values are subjective, arbitrary thresholds and are not to be seen as unerring. With the recommendations of Browne and Cudeck (1993), we applied the following cut off values, for Root Mean Square Error of Approximation (RMSEA): ≤0.05 = close model fit, for the Comparative Fit Index (CFI) and Tucker–Lewis Index (TLI) ≥ 0.80 = acceptable model fit, and >0.90 = good model fit. Tabachnick et al. (2019) suggested thresholds for interpreting factor loadings: ≥0.32 = poor, >0.45 = fair, >0.56 = good >0.63 = very good and >0.71 = excellent. Means and Variance Adjusted Weighted Least Squares (WLSMV) was applied as model estimator as the data was categorical (Brown, 2015).

To examine the differences in Norwegian and Australian PaRCADS scores, we re‐coded the data of the original PaRCADS study, which is openly available online (Sim & Yap, 2018), from binary to continuous scoring. In the original PaRCADS, each response option on a scale item was assigned a score of “0” for non‐adherence and “1” for adherence to the parenting guidelines for the prevention of childhood depression and anxiety (Parenting Strategies Program, 2014). For example, the binary scoring for domain 1, item 1: “almost never = 0, rarely = 0, sometimes = 0, often = 1, almost always = 1” (Sim et al., 2019). In this study, we coded the item scores “almost never = 0, rarely = 1, sometimes = 2, often = 3, almost always = 4”. Negatively worded items were reversely coded. We compared the two datasets through independent samples *t*‐tests.

Three items in PaRCADS apply only to parents who have a partner, we therefore set the nil responses from parents without a partner as system missing in the CFA and internal consistency analyses. Because of this, we multiplied the mean of answered item scores in a domain by the total number of items in each domain to obtain domain scores. We conducted all analyses in SPSS 29, with a macro for calculating Omega (Hayes & Coutts, 2020), except for the CFA, which we analyzed in MPLUS 8.6 (Muthén & Muthén, 1998–2017).

### 2.5 Ethics

The project was approved by the Regional Committees for Medical Health Research Ethics (REK251627).

## 3 RESULTS

### 3.1 Parent and child characteristics

Parent and child demographics are presented in Table 1. In our sample of 163 parents, 80% (*n* = 130) were mothers, with a mean age of 42.2 years (SD = 5.6), their children had a mean age of 9.6 years (SD = 1.3) and 48% were girls (*n* = 78). The proportion of parents with tertiary education (81%) was higher than the same age group in the general Norwegian population (50%) (Statistics Norway, 2023). Fathers (*n* = 30) on average had a PaRCADS total score that was 14 points (95% CI: −23 to −5.8) lower than that of mothers (*n* = 130). Fathers also consistently scored lower in all domains of the PaRCADS. There were no significant correlations between PaRCADS scores and age or education.

**TABLE 1:** Sample characteristics (*N* = 163).

	*n*	%
Parent characteristics		
Age (mean years, SD) (*n* = 160)	42.2 (5.6)	
Relationship to child		
Mother	130	80%
Father	30	18%
Stepparent	3	2%
Nationality		
Norwegian	153	94%
Other (Sweden, Finland, Italy, Poland, Turkey, Ukraine, Hungary)	10	6%
Employment^a^		
Full time employed	131	80%
Part time employed	13	8%
Other		
Stay at home parent	1	1%
Long‐term sick leave	1	1%
Work assessment allowance	5	3%
Disability	9	6%
Studying	5	3%
Completed education		
Lower secondary school	12	7%
Upper secondary school	19	12%
University/university college up to 4 years	34	21%
University/university college more than 4 years	98	60%
Family's financial situation, indicated by self‐evaluation		
Very good	35	22%
Good	94	58%
Mediocre	24	15%
Poor	8	5%
Very poor	2	1%
Child characteristics		
Sex		
Girls	78	48%
Boys	85	52%
Age (mean years, SD)	9.6 (1.3)	
Long term functional impairment/illness (physical or mental)		
No	125	77%
Worried that something is wrong	5	3%
Yes	18	11%
Anxiety or depression	1	1%
Under evaluation	15	9%
Anxiety or depression	6	4%

^a^ One respondent was both studying and receiving work assessment allowance, one respondent was both studying and on disability pension.

Results on General Family Functioning (FAD‐GF), parental warmth (PBI *Warmth* subscale) and child anxiety (SCARED) and depression (SMFQ) symptoms are presented in Table 2. In this sample 25% (*n* = 41) scored 24 or higher on the FAD‐GF scale, indicating that the parent perceives their family functioning as adverse. Nineteen percent scored ≥25 on SCARED, indicating elevated levels of child anxiety symptoms. Twenty‐three percent scored ≥7 on SMFQ, indicating elevated depressive symptoms.

**TABLE 2:** Parent reports on other family scales and parent reported child anxiety and depression scores, mean (SD) and possible range for scales and subscale scores (*N* = 163).

	Range	Total sample	Daughters (*n* = 78)	Sons (*n* = 85)
FAD‐GF	12–48	19.8 (5.0)	19.4 (4.8)	20.2 (5.2)
PBI warmth	7–28	25.0 (2.5)	25.0 (2.4)	25.1 (2.6)
SMFQ	0–26	4.0 (4.3)	4.6 (4.7)	3.5 (3.7)
SCARED	0–82	14.1 (11.2)	15.9 (11.4)	12.4 (10.8)

Abbreviations: FAD, family assessment device; PBI, parental bonding instrument; SCARED, screen for child anxiety related disorders.

### 3.2 Internal consistency

Internal consistency values for all 10 of PaRCADS’ domains, as measured by McDonald's Omega (*ω*) are reported in Table 3, with values from below adequate (*ω* = 0.50) to adequate (*ω* = 0.78).

**TABLE 3:** McDonalds omega (*ω*) for PaRCADS domains (*N* = 163) and Pearson's correlations (*r*) between PaRCADS domain scores and total scores at two time points (test–retest) (*n* = 74).

Domain	*ω*	*r* (95% CI)
1. Parent‐child relationship	0.78	0.70 (0.56–0.80)
2. Involvement in child's life	0.58	0.61 (0.45–0.74)
3. Relationship with others	0.78	0.70 (0.55–0.80)
4. Rules & consequences for child	0.63	0.68 (0.54–0.79)
4. Rules & consequences for child (*n* = 139)^a^	0.69	0.71 (0.57–0.80)
5. Health habits	0.57	0.78 (0.67–0.86)
6. Home environment	0.53	0.71 (0.58–0.81)
6. Home environment (*n* = 138)^a^	0.61	0.71 (0.57–0.81)
7. Managing emotions	0.62	0.70 (0.57–0.80)
8. Setting goals and dealing with problems	0.73	0.65 (0.49–0.76)
9. Dealing with negative emotions	0.60	0.70 (0.56–0.80)
10. Getting help when needed	0.50	0.70 (0.56–0.80)
Total PaRCADS score		0.85 (0.77–0.90)

*Note*: All correlations were significant, *p* < 0.01 (2‐tailed).

Abbreviation: PaRCADS, Parenting to Reduce Child Anxiety and Depression Scale.

^a^ Removed variables with missing values: domain 4 item 7 and domain 6 item 9 and 10, where 24 parents answered “not relevant, I have no partner”.

### 3.3 Test–retest

Pearson correlation coefficients between PaRCADS domains and total scores at the two measurement times are shown in Table 3. On average, participants completed the retest 87 days (SD = 35) after they completed the initial survey. Test–retest reliability for the total score was excellent (*r* = 0.85), and the median stability for the domains was good (*r* = 0.70). There were no substantial difference on group level on parent age, child age, anxiety and depression scores nor proportion of girls, mothers or participants with tertiary education, see supplementary material, Table S1.

### 3.4 Construct validity

Pearson correlation coefficients between PaRCADS domain scores and scores on the FAD‐GF and PBI *Warmth* dimension are reported in Table 4. Except for the *Health habits* domain, the results showed expected positive correlations between PBI *Warmth* dimension and the PaRCADS domains, and PaRCADS total score (*r* = 0.65). As expected, there was a weaker negative correlation between the FAD‐GF score and PaRCADS total score (*r* = 0.47).

**TABLE 4:** Pearson's correlations (*r*) and 95% CI. between PBI Warmth dimension, FAD‐GF and PaRCADS domains (*N* = 163).

PaRCADS domain	PBI warmth		FAD‐GF	
	*r*	95% CI	*r*	95% CI
1. Parent‐child relationship	0.69**	0.61–0.77	−0.44**	0.61–0.77
2. Involvement in child's life	0.46**	0.32–0.57	−0.27**	0.32–0.57
3. Relationship with others	0.46**	0.33–0.58	−0.37**	0.33–0.58
4. Rules & consequences for child	0.52**	0.39–0.62	−0.47**	0.39–0.62
5. Health habits	0.14	−0.01–0.29	−0.09	−0.01–0.29
6. Home environment	0.36**	0.21–0.48	−0.36**	0.21–0.48
7. Managing emotions	0.53**	0.41–0.63	−0.31**	0.41–0.63
8. Setting goals and dealing with problems	0.45**	0.31–0.56	−0.30**	0.31–0.56
9. Dealing with negative emotions	0.49**	0.36–0.60	−0.32**	0.36–0.60
10. Getting help when needed	0.35**	0.21–0.48	−0.26**	0.21–0.48
Total PaRCADS score	0.65**	0.55–0.73	−0.47**	0.55–0.73

Abbreviations: PaRCADS, Parenting to Reduce Child Anxiety and Depression Scale; PBI, Parental Bonding Instrument.

*p < 0.05, **p < 0.01 (2‐tailed).

There were no significant correlations between PaRCADS total scores, and child anxiety and depression symptoms measured by parent‐reports on SCARED and SMFQ. Correlation between PaRCADS and SCARED: *r* = 0.06, 95% CI = −0.10–0.21, *p* = 0.49; correlation between PaRCADS and SMFQ: *r* = −0.05, 95% CI = −0.21–0.10, *p* = 0.50.

### 3.5 Confirmatory factor analysis

**FIGURE 1:**
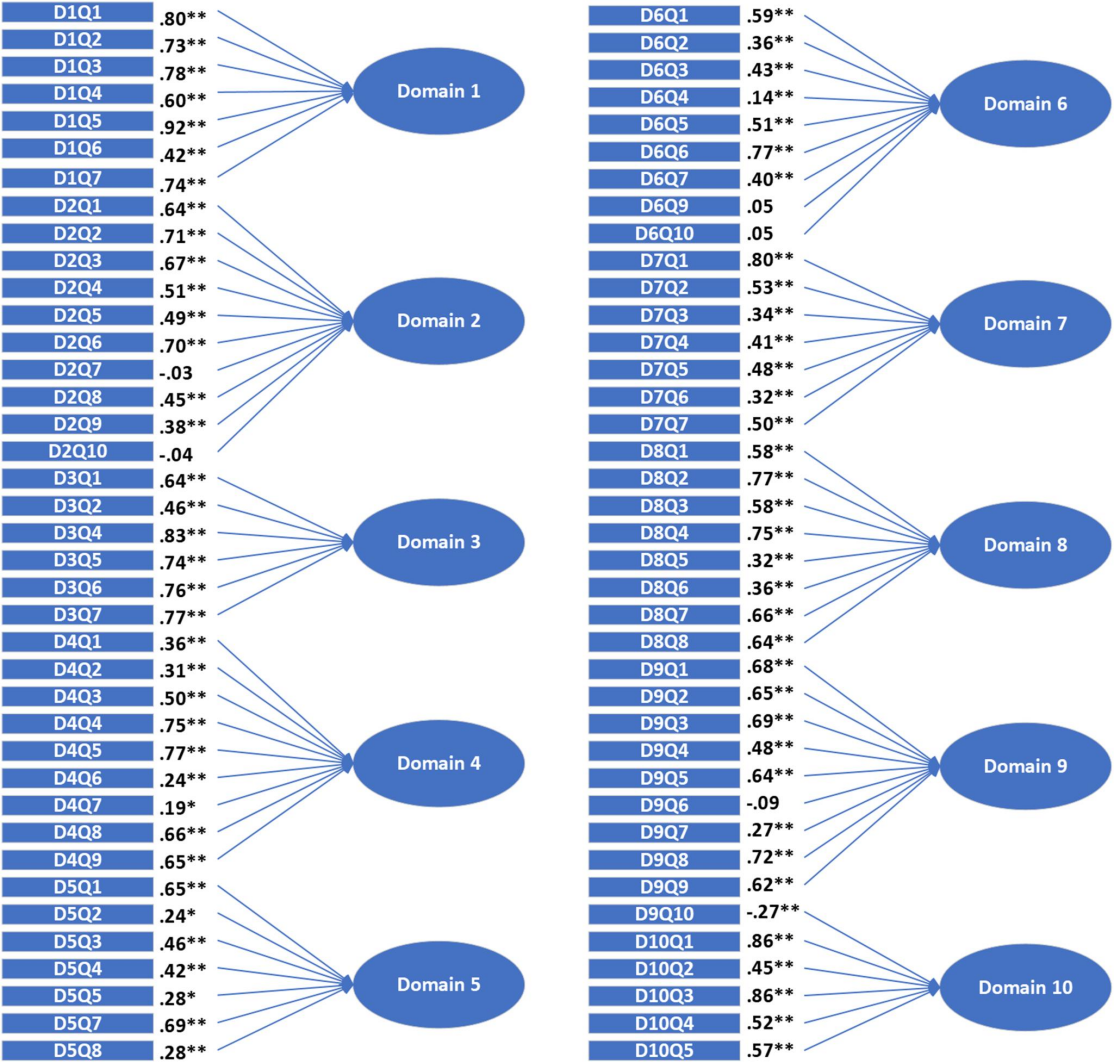
Standard factor loadings of PaRCADS in a Norwegian sample (*N* = 163). PaRCADS dimensions are: 1. Relationship with your child, 2. Involvement in child's life, 3. Child's relationships with others, 4. Rules and consequences, 5. Health habits, 6. Home environment, 7. Managing emotions, 8. Setting goals and dealing with problems, 9. Dealing with negative emotions, 10. Getting help when needed. PaRCADS, Parenting to Reduce Child Anxiety and Depression Scale. *p < 0.05, **p < 0.01 (2‐tailed).

CFA based on the domain structure of PaRCADS as originally suggested by Sim et al. (2019) indicated an acceptable fit between the ten‐factor model and the data (*N* = 163): *χ* ^2^ = 7772.558 (df = 3003), *p* < 0.0001; RMSEA = 0.039; CFI = 0.850 and TLI = 0.844. Standard factor loadings are reported in Figure 1. Domains 1, 3 and 10 performed better, with only significant and fair factor loadings for all but one item in domain 1 (D1Q6).

### 3.6 Comparing PaRCADS scores between the Norwegian and Australian samples

Table 5 shows the comparison of mean scores in the original PaRCADS validation study (Sim et al., 2019) and in the present study, as well as the maximum possible score. The mean total score was significantly higher in the present sample (233.8) than the Australian sample (226.9). There were also significant differences in several of the domain scores, ranging from 0.9 to 2.3 points. The largest difference was seen in the first domain, *Relationship with your child*.

**TABLE 5:** PaRCADS scores, maximum obtainable, mean (SD) and *t*‐test results for scales and subscale scores in the Norwegian and Australian sample.

PaRCADS domain	Max	Mean (SD) score		Mean difference	95% CI
		Norwegian PaRCADS(N) (*N* = 163)	Australian PaRCADS (*N* = 355)		
1. Parent‐child relationship	28	23.1 (3.0)	20.8 (3.1)	2.3**	1.8–2.9
2. Involvement in child's life	40	28.9 (3.2)	28.9 (3.7)	0.0	−0.6–0.7
3. Relationship with others	24	17.6 (3.2)	16.0 (3.3)	1.6**	1.0–2.2
4. Rules & consequences for child	36	24.8 (3.5)	23.1 (4.6)	1.8**	1.0–2.5
5. Health habits	28	21.1 (3.3)	22.1 (3.5)	−0.9**	−1.6 to −0.3
6. Home environment	36	26.7 (3.7)	26.3 (3.9)	0.4	−0.4–1.1
7. Managing emotions	28	20.8 (3.2)	19.8 (3.6)	0.9**	0.3–1.6
8. Setting goals and dealing with problems	32	24.3 (3.6)	23.4 (3.9)	1.0**	0.2–1.7
9. Dealing with negative emotions	40	30.0 (3.5)	29.2 (4.0)	0.7	−0.0–1.4
10. Getting help when needed	20	16.4 (2.3)	17.3 (2.3)	−0.9**	−1.3 to −0.5
Total PaRCADS score	312	233.8 (22.3)	226.9 (24.5)	6.9**	2.4–11.3

Abbreviation: PaRCADS, Parenting to Reduce Child Anxiety and Depression Scale.

**p < 0.01 (2‐tailed).

## 4 DISCUSSION

The primary aim of this study was to investigate the psychometric properties of PaRCADS(N) in a community sample of Norwegian parents, by establishing its reliability, validity, and factor structure. Indices of internal consistency, test–retest reliability, construct validity and factor structure indicate that the psychometric properties of PaRCADS(N) are adequate. We also compared our results to that of the Australian PaRCADS‐study, and examined whether there was a relationship between parental scores on PaRCADS(N) and their child's symptom levels.

Internal consistency values for PaRCADS(N) ranged from inadequate to adequate (*ω* = 0.50 to 0.08), indicating that some of the subscale items are not highly interrelated and might not fully capture the same construct. To our knowledge, this was the first assessment of test–retest reliability of PaRCADS. Pearson's correlations showed that the test–retest reliability for the different domains ranged from adequate (*r* = 0.61) to good (*r* = 0.78) and the test–retest for the total scale was excellent (*r* = 0.85). The lower coefficients for some domains could be explained by the relatively long time between test and retest for some participants due to practical reasons. On average, participants completed the retest 87 days (SD = 35) after the initial test. The response rate of the re‐test was 45%. This response rate gives reason to interpret and generalize these findings with care as we cannot rule out that parents who participated at retest were different from the majority (55%) who did not participate in the retest. However, we found no substantial difference on group level between retest participants and non‐participants on means of the following variables: child age, parent age, anxiety and depression scores nor proportion of girls, mothers or participants with tertiary education. Notwithstanding, these results indicate that PaRCADS(N) is a stable instrument with good test–retest reliability and that PaRCADS(N) may be suited to measure change in parental practices between two time points, for example, before and after interventions for childhood anxiety and depression involving parents.

Construct validity of PaRCADS(N) was indicated by a moderate positive correlation between greater parental warmth as measured by the PBI *Warmth* dimension and PaRCADS(N) total score and a moderate negative correlation between poorer general family functioning, as measured by FAD‐GF and PaRCADS(N) total score. In line with our expectations, there were larger correlations between parental warmth (PBI *Warmth*) and PaRCADS(N) domains pertaining to the parent‐child relationship and parental warmth, such as the *Parent‐child relationship* domain, which had the strongest correlation with PBI *Warmth*. The lowest and only non‐significant correlation with PBI *Warmth* was seen in PaRCADS(N) domain 5, *Health habits*, where we expected a smaller correlation as parental support of good health habits in their child could have minimal overlap with parental warmth. The correlation between general family functioning (FAD‐GF) and PaRCADS(N) total score (*r* = −0.48) was comparable to the results of Sim et al. (2019) (*r* = −0.52). Overall, these patterns of correlations support the construct validity of PaRCADS(N).

PaRCADS was developed to assess parental practices that are associated with childhood anxiety or depression. Four percent of the children (*n* = 7) in this sample were under assessment for or was diagnosed with anxiety or depression. This is comparable to the results of a recent community‐based study in Norway (Morken et al., 2021; Steinsbekk et al., 2022), which supports that the prevalence of anxiety and depression in the current sample adequately reflects those of the general population in this age‐group. Sim et al. (2019) showed a small, but significant negative association between PaRCADS scores and parent reported child anxiety (*r* = −0.14) and depression (*r* = −0.22) as reported by RCADS‐P (Revised Children's Anxiety and Depression Scale, Parent‐report with 25 items). We predicted a small, negative correlation between PaRCADS(N) scores and child anxiety and depression symptoms in this sample. However, we found only small, non‐significant correlations between PaRCADS(N) scores and child anxiety (*r* = 0.06) and depression (*r* = −0.05) scores. It is important to note that child symptoms in the current study were reported by parents, and the relatively low symptom levels could be difficult for parents to reliably detect, as opposed to children with clinical levels of anxiety or depression symptoms. Forty one percent of the Australian sample reported that their child had a past or present mental health or behavioral diagnosis, including, but not limited to anxiety or depression, compared to four percent of the current sample that had received a diagnosis or were under assessment for anxiety or depression. This suggests that a relationship between PaRCADS(N) scores and childhood anxious or depressive problems may be clearer in a clinical group with parents of children with established disorders. The parental risk and protective factors we aim to measure with PaRCADS(N) may manifest itself in child anxious or depressive problems later in the child's life as the children in this study were younger than the mean and median age of onset for these disorders (Solmi et al., 2022). It would therefore be interesting to do a follow‐up study to see if PaRCADS(N) predicts child anxiety or depression symptoms later in life.

PaRCADS was developed as a criterion‐referenced measure related to guidelines for parents to reduce and prevent anxiety or depression disorders in their child. The original authors pointed out that “*the items in each domain were not expected to uniformly represent a single factor*” (Sim et al., 2019, p. 12), therefore, they did not conduct a CFA.

PaRCADS(N) is a comprehensive scale with 78 questions. It provides a unique opportunity to map parental practices related to childhood anxiety and depression in great depth. The output may be used to target relevant risk and protective factors. The use of a single, comprehensive, and coherent parent measure can potentially save time for everyone involved. However, when a child presents to the clinic or community‐based services, such as the school health service, they undergo several assessments which can be time consuming for parents who are already under stress. Thus, there is also a need for short, succinct, user friendly, and sustainable instruments. If the validity and factor structure is strong, one might choose to use certain parental domains to investigate specific parental factors, rather than the full scale, to make the scale more accessible. The CFA results suggest an acceptable to good model fit between the current data and the predefined model. It did, however, reveal several items with poor factor loadings, which indicates that they do not seem to capture the same underlying construct as other items in the same domain. For example, question 10: “*When my child is facing a problem, I try to solve it for him/her*”, in domain 2: “*Involvement in child's life”*, has a small and non‐significant factor loading. Most questions in this domain are positive formulations about how the parent is involved and shows interest in the child, which can serve as a protective factor (Wu & Lee, 2020). Question 10 (which was reversely scored), however, might tap into overinvolvement, which is a different construct, and can present as a risk factor for childhood anxiety (Bayer et al., 2019). The CFA suggests that the overall fit of the model is good, but there is room for improvement with regards to factor structure, and the number of items might be reduced.

Based on the CFA, domains 1 “*Parent‐child relationship*”, 3 “*Relationship with others*”, and 10 “*Getting help when needed*” stands out as having more robust psychometric properties than some of the other domains, which is supported by the test–retest results. In the Parenting Strategies Program (2014), the following guidelines are directly related to these domains. For domain 1: “*Establish and maintain a good relationship with your child*”, by showing affection, taking time to talk and being aware of how to talk about strong emotions and sensitive topics. For domain 3: “*Encourage supportive relationships*”, by encouraging the child to build supportive relationships with extended family, friends and other adults. For domain 10: “*Encourage professional help seeking when needed*” by seeking knowledge about available resources and encouraging professional help seeking when there is a persistent change in mood or behavior. Domains 1 and 3 also showed better construct validity than the other PaRCADS(N) domains. Because of this, we argue that these subscales could be used separately as individual scales. The current results indicate that the factor structure of PaRCADS(N) is adequate, but there is room for improvement.

There was a difference between the mean PaRCADS total score in this sample, and the Australian sample (Sim et al., 2019). This difference was, however, rather small considering the score range, and although significant, it should be interpreted with care. This difference might be due to cultural differences. It is also worth noting, that the study in each country was conducted 5 years apart. The Norwegian sample completed the survey while there were restrictions in place due to the COVID‐19 pandemic. This led to families spending more time together, which may have affected family relationships and interactions. For example, parents in a large Canadian cross‐sectional survey reported more conflicts, but also more positive interactions with their children due to the pandemic (Gadermann et al., 2021). This could have provided opportunities for bonding and motivating families to start adaptive family routines, that extends into everyday life (Evans et al., 2020). Education level and parent age in the two samples were comparable. None of the studies found a significant correlation between parent educational level and PaRCADS score. The Australian study found a small significant correlation between parent age and PaRCADS scores (*r* = 0.10, *p* = 0.05) (Sim et al., 2019), however, this finding was not replicated in the current study.

## 5 STRENGTHS, LIMITATIONS AND FUTURE DIRECTIONS

This study is the first to conduct a CFA and test–retest on PaRCADS, and to investigate the psychometric properties of a comprehensive scale that measures parental factors related to childhood anxiety and depression outside Australia. The sample is thought to be representative for a great part of the parental population in Norway, with regards to the prevalence of childhood anxiety and depression and gender representation, which allows for a degree of generalization of findings. Approximately half of the children were girls (48%), which is a strength as most studies on anxious and depressive problems have a higher proportion of girls in their samples.

The findings reported in this article should be interpreted in the context of the study's limitations. First, this sample was small and consisted mostly of mothers with high socio‐economic status. This warrants cautiousness in generalizing the results. Participants' children had a relatively low level of anxiety and depression symptoms as reported by parents, therefore, the generalizability to a clinical population is limited. However, PaRCADS was not designed specifically for a clinical population, but as a measure to identify parental behaviors to prevent childhood anxiety and depression. Our findings are in line with our expectations and comparable to Sim et al. (2019).

Finally, we investigated the psychometric properties of PaRCADS(N) with norm‐referenced scoring. It would be interesting to test PaRCADS(N) in a clinical sample with higher symptom levels and investigate whether parents of children with anxiety or depression score lower, that is, report less adaptive parental practices. PaRCADS, which was originally developed as a criterion‐referenced scale, has adequate to good psychometric properties when reliability and validity tests for norm‐referenced scales were applied on PaRCADS(N). Future research should examine whether PaRCADS scores are a predictor for offspring's anxiety or depression later in life, in a longitudinal study. Further work on this scale should include a thorough review of the domains with lower internal consistency and items with poor factor loadings. This could lead to a shorter, optimized and more accessible version of the PaRCADS(N) with better psychometric properties.

## AUTHOR CONTRIBUTIONS

**Kristin Ytreland**: Conceptualization; methodology; validation; formal analysis; project administration; writing – review & editing; writing – original draft; investigation; funding acquisition. **Jo Magne Ingul**: Writing – review & editing; methodology; conceptualization; funding acquisition; validation. **Stian Lydersen**: Writing – review & editing; methodology; conceptualization; funding acquisition; validation. **Marie Bee Hui Yap**: Writing – review & editing; conceptualization; validation; methodology. **Wan Hua Sim**: Writing – review & editing; conceptualization; validation; methodology. **Anne Mari Sund**: Writing – review & editing; methodology; conceptualization; funding acquisition; validation. **Elisabeth Valmyr Bania**: Writing – review & editing; methodology; conceptualization; funding acquisition; validation; investigation.

## CONFLICT OF INTEREST STATEMENT

Sim and Yap are authors of the original PaRCADS, but derive no financial benefit from its use or validation in this study. The authors declare that they have no competing interests.

## ETHICS STATEMENT

The authors declare that this work has been conducted in compliance with the ethical standards of the Helsinki Declaration of 1975, as revised in 2008. The study was approved by the Regional Committees for Medical and Health Research Ethics Central Norway (REK‐Central, ref. 251627), and a Data Protection Integrity Act (DPIA) was carried out prior to data collection.

## Supporting information

Supporting Information S1

Supporting Information S2

Table S1

## Data Availability

Research data are not shared.
