# Supplementary material for: Investigating the psychometric properties of PaRCADS—Parenting to Reduce Child Anxiety and Depression Scale in a Norwegian sample
Source: Int J Methods Psychiatr Res. 2024 Mar 9;33(1):e2017. doi: 10.1002/mpr.2017 (PMC10924274; doi:10.1002/mpr.2017)
Supplement: Supplementary file 1 — Supporting Information S1 [file MPR-33-e2017-s003.docx]

The Parenting to Reduce Child Anxiety and Depression Scale (PaRCADS)

# This survey is about your general approach to parenting [Child name]. Please answer the questions as honestly as

# possible.

# 1. Relationship with [Child name]

| **Please indicate how often you do the following:** | | **Almost never** | **Rarely** | **Sometimes** | **Often** | **Almost Always** |
| --- | --- | --- | --- | --- | --- | --- |
| 1.1 | I let [child name] know that I love [him/her]. |  |  |  |  |  |
| 1.2 | I show [child name] physical affection (e.g. hugs, kisses, massages, tickles). |  |  |  |  |  |
| 1.3 | I provide encouragement to [child name] to help [him/her] feel good about  [himself/herself]. |  |  |  |  |  |
| 1.4 | I accept [child name] for who [he/she] is. |  |  |  |  |  |
| 1.5 | I make time to talk to [child name]. |  |  |  |  |  |
| 1.6  **FP** | I have difficulty giving [child name] my full attention when [he/she] wants to talk to me. |  |  |  |  |  |
| 1.7 | I spend quality, one-on-one time with [child name]. |  |  |  |  |  |

# 2. Involvement in [Child name]’s life

| **Please indicate how often you do the following:** | | **Almost never** | **Rarely** | **Sometimes** | **Often** | **Almost Always** |
| --- | --- | --- | --- | --- | --- | --- |
| 2.1 | I do activities together with [child name] that  [he/she] finds fun. |  |  |  |  |  |
| 2.2 | I take time to get to know [child name]'s friends. |  |  |  |  |  |
| 2.3 | I show interest in what [child name] is doing at school. |  |  |  |  |  |
| 2.4 | I monitor [child name]'s performance at school (e.g. check on homework, ask teachers about how [he/she] is doing). |  |  |  |  |  |
| 2.5 | I take steps to limit [child name]’s exposure to age-inappropriate material or any bullying on the internet. |  |  |  |  |  |
| 2.6 | I give [child name] opportunities to do things that [he/she] is good at, so as to build [his/her] confidence. |  |  |  |  |  |
| 2.7  **FP** | I discourage [child name] from trying new things that might make [him/her] feel stressed. |  |  |  |  |  |
| 2.8 | I encourage [child name] to be responsible for some tasks in [his/her] daily life. |  |  |  |  |  |
| 2.9 | I give [child name] the opportunity to lead the way in some activities. |  |  |  |  |  |
| 2.10  **FP** | When [child name] is facing a problem, I try to solve it for [him/her]. |  |  |  |  |  |

# 3. [Child name]'s relationships with others

| **Please indicate how often you do the following:** | | **Almost never** | | **Rarely** | **Sometimes** | **Often** | | **Almost Always** |
| --- | --- | --- | --- | --- | --- | --- | --- | --- |
| 3.1 | [child name] sees me building strong positive relationships with other people. |  |  | |  |  |  | |
| 3.2 | I encourage [child name] to spend time with supportive adults outside the family. |  |  | |  |  |  | |
| 3.4 | I help [child name] develop good social skills. |  |  | |  |  |  | |
| **I provide opportunities for [child name] to:** | | **Almost never** | **Rarely** | | **Sometimes** | **Often** | **Almost Always** | |
| 3.5 | …spend time with supportive extended family members and close family friends. |  |  | |  |  |  | |
| 3.6 | …spend time with [his/her] own friends. |  |  | |  |  |  | |
| 3.7 | …interact with other people in a safe environment, such as a park, or a community club or an organisation. |  |  | |  |  |  | |

4. Rules and consequences

Now we are going to ask you some questions about rules for [child name].

Note: By rules we mean any type of established expectations, limits or guidelines for [child]’s behaviour that [he/she] is aware of.

| **Please indicate to what extent you do the following:** | | | **Yes, for almost everything** | **Yes, for many things** | **Yes, for some things** | **Hardly, only for a few things** | | **No, not at all** |
| --- | --- | --- | --- | --- | --- | --- | --- | --- |
| 4.1 | I have set specific, defined rules for [child name]’s behaviour. | |  |  |  |  |  | |
| 4.2 | I have set specific, defined consequences for times when [he/she] does not follow the rules. | |  |  |  |  |  | |
| 4.3 | [Child name] was involved in developing the rules. | |  |  |  |  |  | |
| 4.4 | I have talked with [him/her] about the reasons behind the rules. | |  |  |  |  |  | |
| 4.5 | I review or adjust the rules to adapt to [child  name]’s maturity and responsibility. | |  |  |  |  |  | |
| **Please indicate how often you do the following:** | | | **Almost never** | **Rarely** | **Sometimes** | **Often** | **Almost Always** | |
| 4.6  **FP** | If [child name] gets upset with me for enforcing consequences, I let [him/her] get away with breaking the rule to keep peace. | |  |  |  |  |  | |
| 4.7  **FP** | My partner and I present different messages and expectations to [child name] regarding rules and discipline. | Not applicable, I don’t have a partner. |  |  |  |  |  | |
| 4.8 | I try to understand [child name]’s perspective when [he/she] feels that a rule or consequence is unfair. | |  |  |  |  |  | |
| 4.9 | I set a good example for [child name] by following rules and laws  myself. | |  |  |  |  |  | |

# 5. Health habits

| **Please indicate how often you do the following:** | | **Almost never** | | **Rarely** | | **Sometimes** | | **Often** | | **Almost Always** |
| --- | --- | --- | --- | --- | --- | --- | --- | --- | --- | --- |
| 5.1 | I encourage [child name] to eat a healthy, balanced diet, including plenty of fresh vegetables and water |  |  | |  | |  | |  | |
| 5.2  **FP** | In my home, treats (e.g. chips, biscuits, chocolates, or soft drinks, etc.) are readily accessible to [child name]. |  |  | |  | |  | |  | |
| 5.3 | I practise good health habits (i.e. healthy diet, regular exercise, good sleep habits). |  |  | |  | |  | |  | |
| 5.4 | I encourage [child name] to sleep and wake up at roughly the same time each day (even on weekends). |  |  | |  | |  | |  | |
| 5.5  **FP** | I allow [child name] to watch TV or use an electronic device in bed before going to sleep. |  |  | |  | |  | |  | |
| 5.7 | I help [child name] to engage in physical activities (e.g. by transporting [him/her] to sports or dance classes, riding a bike or walking with [him/her], encouraging [him/her] to participate in sports at school, etc.). |  |  | |  | |  | |  | |
| 5.8 | I limit the amount of time [child name] spends watching TV or using electronic devices (e.g. the computer, mobile phones and game consoles) at home. |  |  | |  | |  | |  | |

# 6. Home environment

| **Please indicate how often you do the following:** | | | **Almost never** | **Rarely** | **Sometimes** | **Often** | **Almost Always** |
| --- | --- | --- | --- | --- | --- | --- | --- |
| 6.1 | When I have a disagreement with [child name], I discuss the issue and possible solutions with [him/her]. | |  |  |  |  |  |
| 6.2  **FP** | When [child name] misbehaves, I point out [his/her] negative personal qualities (e.g. lazy, spoilt, selfish, etc). | |  |  |  |  |  |
| 6.3 | I encourage [child name] to judge [his/her] actions as ‘good’ or ‘bad’,  rather than judge [himself/herself] as a ‘good’ or ‘bad’ person. | |  |  |  |  |  |
| 6.4  **FP** | When there is a conflict between family members, I encourage everyone to just brush it off and forget about it. | |  |  |  |  |  |
| 6.5 | If I feel angry with others at home, I try to resolve the issue after I have calmed down. | |  |  |  |  |  |
| 6.6 | I help [child name] learn ways to manage conflict, such as through staying calm and listening to others' concerns. | |  |  |  |  |  |
| 6.7 | I discourage family members from putting down or teasing one another. | |  |  |  |  |  |
| ~~6.8~~ | ~~I smack [child name] when I’m angry.~~  *This question is not included in the Norwegian version | |  |  |  |  |  |
| 6.9  **FP** | When I have an argument with my partner, I criticise him/her in a personal way in front of [child name]. | Not applicable, I don’t have a partner. |  |  |  |  |  |
| 6.10  **FP** | When I have an argument with my partner, I get [child name] to be on my side. | Not applicable, I don’t have a partner. |  |  |  |  |  |

# 7. Managing emotions

| **Please indicate how often you do the following:** | | **Almost never** | | **Rarely** | | **Sometimes** | | **Often** | | **Almost Always** |
| --- | --- | --- | --- | --- | --- | --- | --- | --- | --- | --- |
| 7.1 | I teach [child name] ‘feeling words’ by labelling my own and [his/her] emotions as they happen in everyday life. |  |  | |  | |  | |  | |
| 7.2 | I encourage [child name] to maintain a sense of humour in everyday situations. |  |  | |  | |  | |  | |
| 7.3  **FP** | I help [child name] to manage [his/her] strong emotions by encouraging [him/her] to forget about them. |  |  | |  | |  | |  | |
| 7.4  **FP** | When [child name] is upset, I encourage [him/her] to toughen up. |  |  | |  | |  | |  | |
| 7.5 | I monitor movies or news programs that [child name] is exposed to, to see if they could be distressing to [him/her]. |  |  | |  | |  | |  | |
| 7.6  **FP** | When [child name] is feeling angry, I tell [him/her] to stop it. |  |  | |  | |  | |  | |
| 7.7 | I encourage [child name] to have an accepting and realistic view of [himself/herself]. |  |  | |  | |  | |  | |

# 8. Setting goals and dealing with problems

| **Please indicate how often you do the following:** | | **Almost never** | | **Rarely** | | **Sometimes** | | **Often** | | **Almost Always** |
| --- | --- | --- | --- | --- | --- | --- | --- | --- | --- | --- |
| 8.1 | I encourage [child name] to work towards realistic goals and to follow through with them. |  |  | |  | |  | |  | |
| 8.2 | When [child name] gets stuck on a problem, I explore options with [him/her] so that [he/she] can find a way through it. |  |  | |  | |  | |  | |
| 8.3 | When talking with [child name] about a problem [he/she] has dealt with, I praise [his/her] problem-solving efforts, rather than focusing on the outcome. |  |  | |  | |  | |  | |
| 8.4 | I help [child name] learn from [his/her] mistakes. |  |  | |  | |  | |  | |
| 8.5  **FP** | [Child name] sees me giving up on tasks that prove to be too difficult. |  |  | |  | |  | |  | |
| 8.6  **FP** | When [child name] tries a challenging new task or activity and is not at first successful, I encourage [him/her] to move on to something else. |  |  | |  | |  | |  | |
| 8.7 | When [child name] feels discouraged while working towards a goal, I remind [child name] of the times when [he/she] managed to overcome obstacles successfully. |  |  | |  | |  | |  | |
| 8.8 | I encourage [child name] to approach a trusted school staff member about any problems [he/she] has at school. |  |  | |  | |  | |  | |

9. Dealing with negative emotions

The following section includes questions about sadness, anxiety and anger. Everybody experiences these

emotions at times. People may describe sadness, anxiety or anger as feeling “stressed”, “irritable”, “nervous”,

“on edge”, “worried” or “scared”. Although these emotions may be unpleasant, they can be quite useful in

helping us to avoid dangerous situations or solve everyday problems.

| **Please indicate how often you do the following:** | | **Almost never** | | **Rarely** | | **Sometimes** | | **Often** | | **Almost Always** |
| --- | --- | --- | --- | --- | --- | --- | --- | --- | --- | --- |
| 9.1 | I make time to listen and talk with [child name] when [he/she] is upset. |  |  | |  | |  | |  | |
| 9.2 | I ensure that [child name] has sufficient rest and time to do relaxing activities. |  |  | |  | |  | |  | |
| 9.3 | I help [child name] accept that [he/she] can control some things, but not everything that happens in life. |  |  | |  | |  | |  | |
| 9.4  **FP** | When something is bothering [child name], I tell [him/her] that it’s not such a big deal. |  |  | |  | |  | |  | |
| 9.5 | When [child name] is anxious about something, I talk with [him/her] about what might have caused these feelings. |  |  | |  | |  | |  | |
| 9.6  **FP** | When I am struggling with problems in my own life, I turn to [child name] for support. |  |  | |  | |  | |  | |
| 9.7 | I encourage [child name] to face situations that [he/she] is afraid of. |  |  | |  | |  | |  | |
| 9.8 | If [child name] takes steps to manage [his/her] anxiety, I praise [him/her] for doing it. |  |  | |  | |  | |  | |
| 9.9 | I help [child name] learn the strategies that [he/she] can use for calming [himself/herself] down. |  |  | |  | |  | |  | |
| 9.10  **FP** | I step in to help [child name] at the very first sign of stress or anxiety. |  |  | |  | |  | |  | |

# 10. Getting help when needed

| **Please indicate how likely you would be to do the following:** | |  | |  | | |  |  | |  |
| --- | --- | --- | --- | --- | --- | --- | --- | --- | --- | --- |
| **If I noticed a persistent change in [child name]’s mood or behaviour, I would:** | | **Very unlikely** | | **Unlikely** | | | **Neither likely nor unlikely** | **Likely** | | **Very likely** |
| 10.1 | …encourage [child name] to talk to me about what’s going on for [him/her]. |  | |  | |  | |  | |  |
| 10.2  **FP** | …encourage [child name] to get over it and move on. |  | |  | |  | |  | |  |
| 10.3 | …try to determine whether the change in mood or behaviour is caused by a temporary situation, or an ongoing problem. |  | |  | |  | |  | |  |
| 10.4 | …support [child name] in seeking professional help. |  | |  | |  | |  | |  |
| **If I were experiencing problems with depression or anxiety myself, I would:** | | **Very unlikely** | **Unlikely** | | **Neither likely nor unlikely** | | | **Likely** | **Very likely** | |
| 10.5 | …seek professional help. |  | |  | |  | |  | |  |
