# Supplementary material for: Investigating the psychometric properties of PaRCADS—Parenting to Reduce Child Anxiety and Depression Scale in a Norwegian sample
Source: Int J Methods Psychiatr Res. 2024 Mar 9;33(1):e2017. doi: 10.1002/mpr.2017 (PMC10924274; doi:10.1002/mpr.2017)
Supplement: Supplementary file 2 — Supporting Information S2 [file MPR-33-e2017-s002.docx]

**Parenting to Reduce Child Anxiety and Depression Scale (PaRCADS)**

Følgende spørreskjema dreier seg om hvordan du oppdrar barnet ditt. Vennligst svar så ærlig og oppriktig som du kan.

# 1. Forholdet til barnet mitt

| **Vær så snill å kryss av hvor ofte du gjør følgende:** | | **Nesten aldri** | **Sjeldent** | **Noen ganger** | **Ofte** | **Nesten alltid** |
| --- | --- | --- | --- | --- | --- | --- |
| 1.1 | Jeg lar barnet mitt vite at jeg er glad i han/henne |  |  |  |  |  |
| 1.2 | Jeg gir barnet mitt fysiske tegn på kjærlighet (f.eks. klemmer, kiling, klapp på skulderen etc.) |  |  |  |  |  |
| 1.3 | Jeg oppmuntrer barnet mitt til å føle seg bra med seg selv |  |  |  |  |  |
| 1.4 | Jeg anerkjenner barnet mitt som den han/hun er |  |  |  |  |  |
| 1.5 | Jeg tar meg tid til å snakke med barnet mitt |  |  |  |  |  |
| 1.6  **FP** | Det er vanskelig for meg å gi barnet mitt full oppmerksomhet når han/hun ønsker å snakke med meg |  |  |  |  |  |
| 1.7 | Jeg tilbringer kvalitetstid sammen med barnet mitt |  |  |  |  |  |

# 2. Involvering i mitt barns liv

| **Vær så snill å kryss av hvor ofte du gjør følgende:** | | **Nesten aldri** | **Sjeldent** | **Noen ganger** | **Ofte** | **Nesten alltid** |
| --- | --- | --- | --- | --- | --- | --- |
| 2.1 | Jeg gjør aktiviteter sammen med barnet mitt som han/hun synes er gøy |  |  |  |  |  |
| 2.2 | Jeg tar meg tid til å bli kjent med vennene til barnet mitt |  |  |  |  |  |
| 2.3 | Jeg viser interesse for hva barnet mitt gjør på skolen |  |  |  |  |  |
| 2.4 | Jeg følger med på mitt barns skoleprestasjoner (f.eks. sjekker lekser, spør lærere om hvordan det går) |  |  |  |  |  |
| 2.5 | Jeg begrenser mitt barns tilgang til uønsket innhold på internett (f.eks. aldersmessig upassende innhold eller netthets) |  |  |  |  |  |
| 2.6 | Jeg gir barnet mitt mulighet til å gjøre ting han/hun er god til, for å styrke hans/hennes selvtillit |  |  |  |  |  |
| 2.7  **FP** | Jeg fraråder barnet mitt fra å prøve nye ting som kan føre til at han/hun blir stresset |  |  |  |  |  |
| 2.8 | Jeg oppmuntrer barnet mitt til å ta ansvar for noen hverdagslige oppgaver |  |  |  |  |  |
| 2.9 | Jeg gir barnet mitt muligheten til å lede an i noen aktiviteter |  |  |  |  |  |
| 2.10  **FP** | Når barnet mitt står ovenfor et problem forsøker jeg å løse det for han/henne |  |  |  |  |  |

# 3. Mitt barns relasjoner til andre

| **Vær så snill å kryss av hvor ofte du gjør følgende:** | | **Nesten aldri** | | **Sjeldent** | **Noen ganger** | **Ofte** | | **Nesten alltid** |
| --- | --- | --- | --- | --- | --- | --- | --- | --- |
| 3.1 | Barnet mitt ser at jeg bygger sterke, positive relasjoner med andre mennesker |  |  | |  |  |  | |
| 3.2 | Jeg oppmuntrer barnet mitt til å være sammen med støttende voksne utenfor familien |  |  | |  |  |  | |
| 3.4 | Jeg hjelper barnet mitt med å utvikle gode sosiale ferdigheter |  |  | |  |  |  | |
| **Jeg legger til rette for at barnet mitt:** | | **Nesten aldri** | **Sjeldent** | | **Noen ganger** | **Ofte** | **Nesten alltid** | |
| 3.5 | …kan være sammen med støttende slektninger og nære venner av familien |  |  | |  |  |  | |
| 3.6 | …kan være sammen med vennene sine |  |  | |  |  |  | |
| 3.7 | … kan være sammen med andre mennesker i et trygt miljø (f.eks i utelek, besøke venner, idrettslag, annen klubb eller organisasjon) |  |  | |  |  |  | |

# 4. Regler og konsekvenser

Med «regler» menes alle forventninger, grenser eller retningslinjer for oppførsel som barnet kjenner til.

| **Vær så snill å kryss av i hvilken grad du gjør følgende:** | | | **Ja, nesten for alt** | **Ja, for mange ting** | **Ja, for noen ting** | **Knapt, kun for et par ting** | | **Nei, ikke i det hele tatt** |
| --- | --- | --- | --- | --- | --- | --- | --- | --- |
| 4.1 | Jeg har klare regler for oppførselen til barnet mitt | |  |  |  |  |  | |
| 4.2 | Jeg har klare konsekvenser for når barnet mitt ikke følger reglene | |  |  |  |  |  | |
| 4.3 | Barnet mitt er med på å utforme reglene | |  |  |  |  |  | |
| 4.4 | Jeg har snakket med barnet mitt om hvorfor vi har de reglene vi har | |  |  |  |  |  | |
| 4.5 | Jeg tilpasser reglene slik at de passer mitt barns modenhetsnivå og evne til å ta ansvar | |  |  |  |  |  | |
| **Vær så snill og kryss av hvor ofte du gjør følgende:** | | | **Nesten aldri** | **Sjeldent** | **Noen ganger** | **Ofte** | **Nesten alltid** | |
| 4.6  **FP** | Hvis barnet mitt blir opprørt når regelbrudd får konsekvenser lar jeg han/hun slippe unna for husfredens skyld | |  |  |  |  |  | |
| 4.7  **FP** | Partneren min og jeg gir uttrykk for ulike forventninger til barnet når det gjelder regler og konsekvenser | Ikke relevant, jeg har ingen partner |  |  |  |  |  | |
| 4.8 | Jeg forsøker å forstå mitt barns perspektiv når han/hun føler at en regel eller konsekvens er urettferdig | |  |  |  |  |  | |
| 4.9 | Jeg setter et godt eksempel for barnet mitt ved å følge regler og lover selv | |  |  |  |  |  | |

# 5. Helsevaner

| **Vær så snill og kryss av hvor ofte du gjør følgende:** | | **Nesten aldri** | | **Sjeldent** | | **Noen ganger** | | **Ofte** | | **Nesten alltid** |
| --- | --- | --- | --- | --- | --- | --- | --- | --- | --- | --- |
| 5.1 | Jeg oppmuntrer barnet mitt til å spise sunt og variert, inkludert mye fisk, grønnsaker og vann |  |  | |  | |  | |  | |
| 5.2  **FP** | Hjemme hos oss er godteri og snacks lett tilgjengelig for barnet mitt (f.eks. potetgull, sjokolade, brus, saft) |  |  | |  | |  | |  | |
| 5.3 | Jeg praktiserer gode helsevaner (f.eks. spiser sunn mat, regelmessig trening og gode søvnvaner) |  |  | |  | |  | |  | |
| 5.4 | Jeg oppmuntrer barnet mitt til å sovne og våkne på omtrent samme tid hver dag, også i helgene |  |  | |  | |  | |  | |
| 5.5  **FP** | Jeg lar barnet mitt se på TV eller bruke PC, nettbrett eller mobil i sengen før han/hun sovner |  |  | |  | |  | |  | |
| 5.7 | Jeg legger til rette for at barnet mitt deltar i fysisk aktivitet, f.eks. ved å kjøre barnet til/fra slike aktiviteter, sykle eller gå sammen med barnet og oppmuntre til å delta i idrettsaktiviteter |  |  | |  | |  | |  | |
| 5.8 | Jeg begrenser skjermtid for barnet mitt hjemme (f.eks. bruk av mobil, nettbrett, PC eller TV) |  |  | |  | |  | |  | |

# 6. Hjemmemiljø

| **Vær så snill å kryss av i hvilken grad du gjør følgende:** | | | **Ja, nesten for alt** | **Ja, for mange ting** | **Ja, for noen ting** | **Knapt, kun for et par ting** | **Nei, ikke i det hele tatt** |
| --- | --- | --- | --- | --- | --- | --- | --- |
| 6.1 | Når barnet mitt og jeg er uenige diskuterer vi problemet og mulige løsninger sammen | |  |  |  |  |  |
| 6.2  **FP** | Når barnet mitt oppfører seg dårlig påpeker jeg hans/hennes dårlige personlige egenskaper (f.eks. lat, bortskjemt, egoistisk etc.) | |  |  |  |  |  |
| 6.3 | Jeg oppmuntrer barnet mitt til å vurdere sine handlinger som «gode» eller «dårlige» heller enn å se seg selv som et «godt» eller «dårlig» menneske | |  |  |  |  |  |
| 6.4  **FP** | Når det er en konflikt mellom familiemedlemmer oppmuntrer jeg alle til å børste det av seg og glemme det | |  |  |  |  |  |
| 6.5 | Hvis jeg er sint på noen andre hjemme forsøker jeg å løse problemet etter at jeg har roet meg ned | |  |  |  |  |  |
| 6.6 | Jeg hjelper barnet mitt å lære seg å håndtere konflikter f.eks. ved å bevare roen og å lytte til andres synspunkter | |  |  |  |  |  |
| 6.7 | Jeg fraråder familiemedlemmer å erte eller å trykke hverandre ned | |  |  |  |  |  |
| ~~6.8~~ | ~~Jeg slår barnet mitt når jeg er sint~~  *Dette spørsmålet går ut i norsk versjon/This question is not in the Norwegian version | |  |  |  |  |  |
| 6.9  **FP** | Når jeg krangler med partneren min kritiserer jeg han/henne foran barnet mitt | Ikke relevant, jeg har ingen partner |  |  |  |  |  |
| 6.10  **FP** | Når jeg krangler med partneren min får jeg barnet mitt til å ta min side. | Ikke relevant, jeg har ingen partner |  |  |  |  |  |

# 7. Håndtere følelser

| **Vær så snill å kryss av hvor ofte du gjør følgende:** | | **Nesten aldri** | | **Sjeldent** | | **Noen ganger** | | **Ofte** | | **Nesten alltid** |
| --- | --- | --- | --- | --- | --- | --- | --- | --- | --- | --- |
| 7.1 | Jeg lærer barnet ord om følelser gjennom å sette navn på mine egne og hans/ hennes følelser etter hvert som de oppstår i hverdagen |  |  | |  | |  | |  | |
| 7.2 | Jeg oppmuntrer barnet mitt til å ha en humoristisk sans i hverdagslige situasjoner |  |  | |  | |  | |  | |
| 7.3  **FP** | Jeg hjelper barnet mitt med å håndtere hans/hennes sterke følelser ved å oppmuntre han/henne til å glemme dem |  |  | |  | |  | |  | |
| 7.4  **FP** | Når barnet mitt er opprørt, oppfordrer jeg han/henne til å ta seg sammen |  |  | |  | |  | |  | |
| 7.5 | Jeg følger med på hvilke filmer eller nyheter barnet mitt ser på, for å vurdere om de kan oppleves urovekkende for han/henne |  |  | |  | |  | |  | |
| 7.6  **FP** | Når barnet mitt er sint ber jeg han/henne om å slutte med det |  |  | |  | |  | |  | |
| 7.7 | Jeg oppmuntrer barnet mitt til å ha et aksepterende og realistisk syn på seg selv |  |  | |  | |  | |  | |

# 8. Sette mål og håndtere problemer

| **Vær så snill å kryss av hvor ofte du gjør følgende:** | | **Nesten aldri** | | **Sjeldent** | | **Noen ganger** | | **Ofte** | | **Nesten alltid** |
| --- | --- | --- | --- | --- | --- | --- | --- | --- | --- | --- |
| 8.1 | Jeg oppmuntrer barnet mitt til å jobbe mot realistiske mål og til å forsøke å nå dem |  |  | |  | |  | |  | |
| 8.2 | Når barnet mitt strever med et problem, utforsker jeg alternativer sammen med han/henne for at han/hun skal kunne finne løsninger |  |  | |  | |  | |  | |
| 8.3 | Når jeg snakker med barnet mitt om et problem han/hun har taklet, gir jeg skryt for hans/hennes forsøk på problemløsning, i stedet for å fokusere på resultatet |  |  | |  | |  | |  | |
| 8.4 | Jeg hjelper barnet mitt med å lære av sine feil |  |  | |  | |  | |  | |
| 8.5  **FP** | Barnet mitt ser at jeg gir opp oppgaver som viser seg å være for vanskelige |  |  | |  | |  | |  | |
| 8.6  **FP** | Når barnet mitt prøver seg på en utfordrende ny oppgave eller aktivitet og ikke lykkes på første forsøk, oppmuntrer jeg han/henne til å gjøre noe annet isteden |  |  | |  | |  | |  | |
| 8.7 | Når barnet mitt føler seg motløs i arbeidet mot et mål, minner jeg om når han/hun har vunnet over hindringer tidligere |  |  | |  | |  | |  | |
| 8.8 | Ved problemer på skolen oppmuntrer jeg barnet mitt til å kontakte en ansatt som han/hun har tillit til |  |  | |  | |  | |  | |

# 9. Håndtere negative følelser

De følgende spørsmålene handler om tristhet, engstelse og sinne. Alle opplever disse følelsene innimellom. En kan beskrive *tristhet, engstelse* eller *sinne* som å være *stresset*, *irritabel*, *nervøs*, *bekymret* eller *redd*. Selv om disse følelsene kan oppleves som ubehagelige kan de være svært nyttige for at vi skal unngå farlige situasjoner eller løse hverdagsproblemer

| **Vær så snill å kryss av hvor ofte du gjør følgende:** | | **Nesten aldri** | | **Sjeldent** | | **Noen ganger** | | **Ofte** | | **Nesten alltid** |
| --- | --- | --- | --- | --- | --- | --- | --- | --- | --- | --- |
| 9.1 | Jeg tar meg tid til å lytte og snakke med barnet mitt når han/hun er opprørt |  |  | |  | |  | |  | |
| 9.2 | Jeg passer på at barnet mitt får tilstrekkelig tid til å hvile og til å gjøre noe som er avslappende for han/henne |  |  | |  | |  | |  | |
| 9.3 | Jeg hjelper barnet mitt med å akseptere at han/hun kan kontrollere noe, men ikke alt som skjer i livet |  |  | |  | |  | |  | |
| 9.4  **FP** | Når noe plager barnet mitt, forteller jeg han/henne at det ikke er noe å ta på vei for |  |  | |  | |  | |  | |
| 9.5 | Når barnet mitt er engstelig for noe, snakker jeg med han/henne om hva som kan være grunnen for denne følelsen |  |  | |  | |  | |  | |
| 9.6  **FP** | Når jeg strever med problemer i mitt eget liv, søker jeg støtte hos barnet mitt |  |  | |  | |  | |  | |
| 9.7 | Jeg oppmuntrer barnet mitt til å møte situasjoner som han/hun er redd for |  |  | |  | |  | |  | |
| 9.8 | Hvis barnet mitt gjør noe for å håndtere angsten sin, skryter jeg av han/henne for det |  |  | |  | |  | |  | |
| 9.9 | Jeg hjelper barnet mitt å lære strategier som han/hun kan bruke for å roe seg ned |  |  | |  | |  | |  | |
| 9.10  **FP** | Så snart barnet mitt viser tegn på stress eller angst griper jeg inn for å hjelpe |  |  | |  | |  | |  | |

# 10 Skaffe hjelp ved behov

| **Vær så snill å kryss av i hvilken grad det er sannsynlig at du gjør følgende** | |  | |  | | |  |  | |  |
| --- | --- | --- | --- | --- | --- | --- | --- | --- | --- | --- |
| **Dersom jeg skulle oppfatte en vedvarende endring i humøret eller oppførselen til barnet mitt ville jeg:** | | **Veldig usannsynlig** | | **Usannsynlig** | | | **Verken eller** | **Sannsynlig** | | **Veldig sannsynlig** |
| 10.1 | … oppmuntret han/henne til å snakke med meg om hva som skjer |  | |  | |  | |  | |  |
| 10.2  **FP** | …oppmuntret han/henne til å komme seg over det og gå videre |  | |  | |  | |  | |  |
| 10.3 | … forsøkt å finne ut av om hans/hennes humør skyldes en midlertidig situasjon eller et vedvarende problem |  | |  | |  | |  | |  |
| 10.4 | … hjulpet han/henne med å søke profesjonell hjelp |  | |  | |  | |  | |  |
| **Dersom jeg selv skulle oppleve problemer med depresjon eller angst ville jeg:** | | **Veldig usannsynlig** | **Usannsynlig** | | **Verken eller** | | | **Sannsynlig** | **Veldig sannsynlig** | |
| 10.5 | …søkt profesjonell hjelp |  | |  | |  | |  | |  |
