# Supplementary material for: Investigating the psychometric properties of PaRCADS—Parenting to Reduce Child Anxiety and Depression Scale in a Norwegian sample
Source: Int J Methods Psychiatr Res. 2024 Mar 9;33(1):e2017. doi: 10.1002/mpr.2017 (PMC10924274; doi:10.1002/mpr.2017)
Supplement: Supplementary file 3 — Table S1 [file MPR-33-e2017-s001.docx]

**Table S1:** Descriptive data on participants and non-participants at retest as well as parent-reported anxiety (SCARED) and depression (SMFQ) scores.

| **Variable** | **Participants at initial test (N=163)** | **Participants at retest (n=75)** | **Non-participants at retest (n=88)** |
| --- | --- | --- | --- |
| Child age, mean (SD) | 9.6 (1.3) | 9.7 (1.2) | 9.6 (1.3) |
| Parent age, mean (SD) | 42.2 (5.6) | 42.9 (5.5) | 41.5 (5.6) |
| Anxiety (SCARED) score, mean (SD) | 14.1 (11.2) | 15.0 (11.4) | 13.4 (11.0) |
| Depression (SMFQ) score, mean (SD) | 4.0 (4.2) | 4.2 (4.0) | 3.9 (4.5) |
| Girls, proportion | 48% | 41% | 53% |
| Mothers, proportion | 80% | 79% | 81% |
| Tertiary education, proportion | 81% | 84% | 78% |
